# What I make up when I wake up: anti-experience views and narrative fabrication of dreams

**DOI:** 10.3389/fpsyg.2013.00514

**Published:** 2013-08-13

**Authors:** Melanie G. Rosen

**Affiliations:** Australian Research Council Centre of Excellence in Cognition and its Disorders, Macquarie UniversitySydney, NSW, Australia

**Keywords:** dreaming, fabrication, skepticism, philosophy, epistemology, memory

## Abstract

I propose a narrative fabrication thesis of dream reports, according to which dream reports are often not accurate representations of experiences that occur during sleep. I begin with an overview of anti-experience theses of Norman Malcolm and Daniel Dennett who reject the received view of dreams, that dreams are experiences we have during sleep which are reported upon waking. Although rejection of the first claim of the received view, that dreams are experiences that occur during sleep, is implausible, I evaluate in more detail the second assumption of the received view, that dream reports are generally accurate. I then propose a “narrative fabrication” view of dreams as an alternative to the received view. Dream reports are often confabulated or fabricated because of poor memory, bizarre dream content, and cognitive deficits. It is well documented that narratives can be altered between initial rapid eye movement sleep awakenings and subsequent reports. I argue that we have reason to suspect that initial reports are prone to inaccuracy. Experiments demonstrate that subjects rationalize strange elements in narratives, leaving out supernatural or bizarre components when reporting waking memories of stories. Inaccuracies in dream reports are exacerbated by rapid memory loss and bizarre dream content. Waking memory is a process of reconstruction and blending of elements, but unlike waking memory, we cannot reality-test for dream memories. Dream experiences involve imaginative elements, and dream content cannot be verified with external evidence. Some dreams may involve wake-like higher cognitive functions, such as lucid dreams. Such dreams are more likely to elicit accurate reports than cognitively deficient dreams. However, dream reports are generally less accurate than waking reports. I then propose methods which could verify the narrative fabrication view, and argue that although the theory cannot be tested with current methods, new techniques and technologies may be able to do so in the future.

## INTRODUCTION

The “received view” of dreams is that dreams are conscious experiences we have during sleep which are sometimes reported upon waking. This can be separated into two claims: (1) dreams are experiences that occur during sleep and (2) dreams are reported upon waking. This view is not only widely accepted in the literature^1^, but it is also the common sense view of anyone who has remembered a dream. Challenges to this view rarely surface, although here I evaluate and highlight some of its weaknesses. Norman Malcolm and Daniel Dennett propose *anti-experience *views, they deny the first claim of the received view. Although I highlight evidence which demonstrates that conscious experience does occur during sleep, Malcolm and Dennett raise important issues that are not explained by the received view. I show that a rejection of the *second *claim of the received view provides a more plausible explanation of the issues raised by Malcolm and Dennett. I propose that dream reports are not always reports of experiences we have during sleep, but rather are often fabricated or confabulated narratives created by the waking mind. I refer to this as the *narrative fabrication thesis*. This type of fabrication is itself an interesting phenomenon worthy of detailed analysis. Although it may be possible for some dreams to be accurately reported, given our current technology and methods it is very difficult to discern whether any particular dream report is an accurate representation of the dream content, and there is no fail-safe method to ensure accuracy. I will propose future methods that could be used to test the extent to which narrative fabrication occurs. I begin with an overview Malcolm’s and Dennett’s views, highlighting their various strengths and weaknesses.

## CHALLENGES TO THE RECEIVED VIEW

Malcolm (1956, 1957, 1959) argues that dreams are logically dependent on waking reports. According to Malcolm, “the concept of dreaming is derived, not from dreaming, but from descriptions of dreams” (Malcolm, 1959, p. 55). We cannot verify whether experiences occur during sleep, all that we know is that dream reports are made upon waking.

Malcolm’s argument heavily relies on his criteria for being “sound asleep.” For sleep to fall under “the normal criteria [...] in the ordinary sense” (Malcolm, 1959, p. 30), a sleeping individual must be *prone*, *unaware* of their surroundings and *unresponsive* to stimuli; if someone responds to the question “are you asleep?,” any response would prove that they are *awake*. People who sleep talk and sleep walk therefore are not genuinely sound asleep. They involve “qualified assertions” (p. 29) about non-stereotypical sleeping states. To assert that something occurred in a dream is to assert that it did not *really* happen^2^. Asserting “I am sound sleep” is contradictory. Malcolm infers that I cannot *judge* that I am asleep, since to be able to judge what state I am in, I must first be given an ostensive definition and learn how to appropriately apply the phrase “I am in this state.” This cannot be done whilst asleep. Malcolm is a strong supporter of Wittgenstein’s view that the concepts expressed by language must not be derived from purely subjective experience. “If language is to be a means of communication there must be agreement not only in definitions but also […] in judgments” (Wittgenstein, 1958, p. 242). It is the intersubjective practice of discourse during waking life that gives intelligibility to language, and such discourse cannot occur during sleep.

Judging “I *was* dreaming” relies on a waking inference about a previous state. We cannot guarantee that a dreamer’s judgment “I had a dream” refers to a sleeping experience as there was no behavior at the time to verify the claim. This distinguishes the dream report from a waking state report such as “I was in pain” since there may have been pain behavior during the waking pain state. Since we are unable to judge when we are asleep, and unable to judge when another is dreaming, Malcolm concludes that sleep experiences are unverifiable. We derive our concept of dreaming not from experiences that occur during sleep, but from the reports we make upon waking. However, if experience does not occur during sleep, Malcolm’s view does not explain why we report dreams.

Dennett (1976) regards Malcolm’s verificationist approach as “too drastic” (p. 159), although he also finds the received view to be problematic. Rather than simply attempting to undermine the authority of the received view, Dennett goes a step further than Malcolm by proposing potential rivals. One such rival is the *cassette view, *that dreams are memories that are inserted into consciousness like cassettes upon waking up.

Dennett discusses a type of dream that provides evidence against the received view which I call the *suspense dream.* In such a dream, suspense builds up to an event that coincides with some sensory stimulus from the external world^3^. In an anecdotal example, the dreamer is in a saloon and is challenged to a duel. The dreamer is shot, and the sound of the gun coincides with a car backfiring in the external world. Dennett himself has experienced such dreams, and they have been reported in the scientific literature. In one experiment, water was dripped on a subject’s back during rapid eye movements (REM) sleep. Upon waking the subject reported dreaming of singing in an opera, then seeing the soprano being struck by falling water. When he ran to her and bent down, he felt dripping water on his back (Dennett, 1976, p. 158). It is likely that in these examples, the stimulus has filtered into the dream, causing the dreamer to hear a bang or feel dripping water. The unusual build-up towards this event, and then the seamless incorporation of the sound into the narrative, as if scripted, suggests the dream is precognitive, that it predicts the car backfiring or water dripping. A dreamer hearing a bang and turning around to see that a gun went off is not problematic for the received view since there is no element of suspense. Dennett argues that if the received view is correct, we must attribute some type of prescience to the dreamer. Since precognition is dreams is highly improbable, the received view is implausible.

Dennett admits that most reports of suspense dreams are anecdotal, and although there are some noted in the literature, they are not commonly documented. If they occur statistically rarely, the suspense element could be due to coincidence, or perhaps dream narratives are sufficiently flexible to seamlessly incorporate a variety of external stimuli. Both explanations are consistent with the received view, but Dennett rejects them. He notes that together the anecdotal examples and examples from experiments are sufficiently common to rule out coincidence, and the cases in the literature do not support the flexible narrative explanation.

Dennett focuses on an alternative to the received view called the cassette view: memories, like cassettes, are inserted into consciousness upon awakening. These memories are stored in our minds like a library of undreamed dreams and have various endings that can be replayed in response to relevant external stimuli. When we wake up, we simply *replay* these “cassettes” as if remembering an experience that had just occurred whilst asleep. Generation of the cassettes could occur during REM sleep, which would explain the increased brain activation. Dennett notes that cassette generation is difficult to explain, yet there has been no widely accepted explanation of dream generation according to the received view either. This is still true today. Under this view lucid dreaming is not awareness of dreaming, but rather a sense when we wake up that the generation of the cassette had an element of conscious intention. Dream “memories” are false memories.

Many theorists find this skepticism about dreaming implausible^4^, as it contradicts current empirical evidence. I agree that it is implausible to deny that conscious experience occurs during sleep. I instead propose a more plausible challenge to the received view that is both consistent with the current empirical data and that solves the problems raised by Malcolm and Dennett.

## SLEEP AS DEFINED BY NEUROPSYCHOLOGY AND LUCID DREAMING CONFIRM THAT EXPERIENCE OCCURS DURING SLEEP

Revonsuo (1995) contends that Malcolm’s argument fails due to its reliance on faulty assumptions about sleep. Stages of sleep are defined by *neuropsychology*: different stages of sleep are classified according to *brain activity* rather than behavior or lack thereof. Although REM sleep typically causes paralysis to the body, abnormalities such as REM sleep behavior disorder (RBD) can occur (Windt and Metzinger, 2007, p. 5) in which the dreamer appears to act out their dreams. Experiments have correlated dream reports with RBD behavior (Schenck et al., 1987). Scientists consider abnormal cases such as RBD to be under the criterion of REM sleep. Contrary to Malcolm’s view, an individual may be asleep despite sitting up in bed and acting as if they are hallucinating. Since his definition of being sound asleep plays such an important role in Malcolm’s argument, the possibility that an individual could act out their dream provides strong evidence against his view. However, since Dennett’s view does not rely on Malcolm’s definition of being “sound asleep,” his view is not weakened by this evidence. Appearing to act out a dream may not mean that a subject is consciously aware of dreaming, but might be an unconscious response to the “cassette production” that occurs during REM sleep.

Eye signaling experiments on lucid dreamers provide sufficient evidence to disprove the anti-experience thesis. Lucid dreamers can learn to signal that they are dreaming by performing a series of learned eye movements (LaBerge, 1981). I can see no other plausible explanation for this ability than that experience occurs during sleep, and that the dreamer is aware they are dreaming. One could argue that the eye movements are an automatic response after they had been learned and rehearsed sufficiently. However, this seems implausible since they correlate *only* with lucid dream reports and an automatic response could occur during any session of sleep. Although lucid dream signaling cannot verify non-lucid dream experience, lucid dreams provide strong evidence that conscious experience does occur during sleep. Dennett’s explanation of lucid dreams is implausible. If lucidity is merely a sense of agency over cassette generation, it is unclear how an individual could gain sufficient self-awareness whilst asleep to carry out pre-learned eye movements. If I learn to flick my eyes a certain way when I realize I am dreaming, although this may not be *literally* asserting “I am dreaming,” this is a communication of “I am dreaming” which is sufficient to verify a dream is occurring. So Malcolm’s argument that I could never learn to appropriately assert “I am dreaming” is a red herring, since I can certainly learn to *communicate* “I am dreaming” whilst dreaming using eye flicks.

I conclude that Malcolm’s definition of being sound asleep is highly implausible given current data on sleep stages. A sleeping individual may, for example, exhibit pain behavior and still be “sound asleep.” The strongest evidence that experience occurs during sleep is lucid dreaming experiments that involve a participant signaling that they have gained lucidity. This cannot be plausibly explained by either Malcolm’s or Dennett’s views. Nonetheless, as I show now, the received view of dreams is flawed, and an alternative explanation for certain problematic phenomena is required. I provide an explanation which is more plausible than Malcolm’s and Dennett’s views.

## ROOM FOR SKEPTICISM: WHAT THE RECEIVED VIEW DOES NOT EXPLAIN

The received view of dreaming as of yet does not explain some of the empirical data and issues raised by Malcolm’s and Dennett’s views. In this section I will discuss why there is room to be skeptical of the received view even if we reject Malcolm’s and Dennett’s anti-experience arguments and accept that experiences do occur during sleep.

Firstly, the received view does not explain why eye movements and other sleep behaviors are difficult to correlate with non-lucid dream reports. The discovery of REM sleep led Dement and Kleitman (1957a,b) to propose a *scanning hypothesis *of dreams: that REMs scan dream imagery. Early experiments attempted to demonstrate that dream reports are highly correlated with eye movements, for example, dreams about throwing a basketball into a hoop correlate with vertical eye movements. However, these experiments were based on small sample sizes and the results have not been replicated in subsequent experiments. Doricchi et al. (2006) claim that “despite decades of research, the question of whether the REMs of paradoxical sleep (PS) are equivalent to waking saccades and whether their direction is congruent with visual spatial events in the dream scene is still very controversial”. According to Hobson et al. (2000), “further evidence would be required to confirm [the scanning] hypothesis”. The strongest correlations made to date are from lucid dream experiments^5^. Lucid dreamers can carry out specific, prearranged tasks during a lucid dream and signal whilst they are performing these tasks using eye-flicks and muscle twitches (Schatzman et al., 1988) which correlate with dream reports. However, lucid dreaming is not indicative of all sleep phenomena and it is difficult to verify non-lucid dream content. Although lucid dreaming in particular provides strong evidence that experience does occur during sleep, if the received view were correct, we would expect either that correlations between reports and eye-movements could either be made in both lucid and non-lucid dreams, or in neither^6^. However, as I argue my view has a plausible explanation as to why such correlations are so tenuous in non-lucid dreams.

Foulkes (1999, p. 10) notes that other sleep behavior such as sleep walking rarely corresponds to dream reports and “there is a somewhat better, but still imperfect, relation between sleep speech and dream speech.” Arkin et al. (1966,1970a,b), argue that they demonstrate significant overlap between dream reports and sleep talk that occurred during REM sleep, however, this is not clearly the case. Arkin et al. (1966) examined the dream reports and sleep talk of a habitual sleep talker (S) to find correlations between the two. They found that higher rates of sleep talk could be invoked by hypnotizing S before he fell asleep. They used post-hypnotic suggestion (PHS) “tonight you will sleep normally and naturally and talk in your sleep in the same manner as you do at home, but more abundantly” (p. 295). Electro-oculogram (EOG) and Electroencephalogram (EEG)^7^ were used to compare eye movements and brain activity to verify during which sleep stages the talking occurred. The content of the sleep talking sessions, according to Arkin and colleagues, correlated strongly with the content of the dream reports collected upon waking. For example, sleep talk:

Now viewing a film of past experiences in gallery for small admission charge (Arkin et al., 1966, p. 305) correlated with the dream report:

Uh-[pause]-there’s a theatre that you pay admission charge and they run films of your life-that’s all I remember except that I was in one of those theatres a minute ago (Arkin et al., 1966, p. 305).

Arkin and colleagues also found that most aspects of hypnotized sleep replicate non-hypnotized sleep, with similar electrical brain activity and similar but somewhat gentler eye movements. This might provide evidence that non-hypnotized sleep speech is also associated with dream content, but there are some concerns regarding these studies. Firstly, correlations between reports and sleep talk in this study were often vague. Unclear, mumbled words are often assumed to be words that correlate with the report, but this is not always justified^8^. As I discuss in Section “Confabulation and Memory Loss,” some reports suggest the opposite, that dream reports are not a good indication of dream content. Secondly, it is not clear whether hypnotism affects the content of dreams or the accuracy of the report. Waking memory reports involving hypnotism are subject to increased confabulation. Ofshe (1992) found that hypnotism can cause a subject to be susceptible to suggestion, or even cause false memories. Therefore dream memories after hypnotism may also be false. The received view does not explain why reliable correlations between sleep talk and dream reports are so elusive.

Finally an important point raised by Malcolm is that we should realize the limitations of neuroimaging in providing information about cognitive states. Neuroimaging provides information about activity in the brain whilst asleep and data about the activation of specific brain regions can then be correlated with reports. Hobson et al. (2000) and other theorists use neuroimaging data to correlate dream reports with neural activation under the assumption that neuroimages can help verify the content of dream reports. Schwartz and Maquet (2002) note that “the specific regional distribution of brain activity during REM sleep might [...] be linked to specific dream features” (Schwartz and Maquet, 2002, p. 23). Malcolm (1959) rejects this possibility, arguing that we cannot infer experience from brain activation. We could not infer that a certain pattern of activation that correlates with conscious experience whilst awake would correlate with the same experience during sleep. Brain activation without a subjective report is not sufficient to verify a conscious state is occurring. This view has recently gained supported from cognitive neuroscientists.

Klein (2010a,b) notes that making inferences from images of brain function to psychological function can be problematic for many reasons. One problem is that the brain is a densely connected system and any cognitive task should cause increased activation all over the brain. Functional magnetic resonance imaging (fMRI) imaging uses a threshold to distinguish between relevant and irrelevant activation, however, the choice of threshold is an arbitrary decision made by the experimenter, and this choice can have theoretically important implications for the research (Klein, 2010a). Secondly, inferences made about the psychological function of a type of brain activation are already biased towards the preconceived notions of the experimenter^9^. A more extreme view proposed by Harley (2004) and Coltheart (2006), is that neuroimaging data are *irrelevant* to psychology because we do not yet have sufficiently precise theories about how psychology relates to particular neural phenomena. Theories about the relation between particular brain activity and psychology are required before neuroimaging can tell us anything about psychology, i.e., by giving predictions about psychological events. Yet once each theory is formulated and confirmed, “[neuroimaging] would have nothing of interest to contribute to psychology” (Klein, 2010b, p. 169) since all of the important information would already be known^10^.

With current technology, it is impossible to discern the specifics of dream content, such as characters, and events. For example, there is no way to discern that a dream is about playing with puppies from brain activation. Although neural activation correlated with reports gives *some *evidence that consciousness occurs during sleep, distinguishing between consciousness and unconsciousness using neuroimaging data alone can be difficult. Monti (2010) notes that misdiagnoses of the conscious state of vegetative patients occurs in around 40% of cases. In sleeping subjects, this is complicated by the “input blockade” (Hobson, 2002), which means that in most cases, subjects are unresponsive to external stimuli. Verifying whether a subject is conscious or aware of internally generated dream stimuli is still primarily dependent on dream reports. Levy’s (2006) discussion of research on unconscious patients who exhibit complex unconscious cognitive processing demonstrates that complex cognitive processing can occur when a subject is unconscious. In one experiment, unconscious patients in a persisting vegetative state exhibited brain behavior suggesting that they can distinguish between sentences with congruent and incongruent endings, a complex task that theorists previously thought could not be completed unconsciously. This is why I believe that lucid dream signaling, rather than neuroimaging, provides the strongest evidence that consciousness occurs during sleep. The upshot of this is that neuroimaging, using current technology and methods, cannot confirm the second claim of the received view, that dream are accurately reported upon waking.

The received view fails to provide a convincing explanation of suspense dreams, the failure of research groups to either confirm or deny the scanning hypothesis and finally, why correlations between sleep talk and sleep reports are so tenuous. Furthermore, the second claim of the received view, that dream reports are accurate, is not verified by evidence from neuroimaging. I now propose a view that provides a plausible explanation for these findings.

## NARRATIVE FABRICATION IN DREAM REPORTS

Since the received view of dreams does not explain why correlations between eye movements and dream reports are so tenuous, why sleep behavior and sleep talk are so rarely correlated with dream reports, or suspense dreams, I propose a view that takes these failings into account. Although I have argued that it is implausible to deny the first claim the received view, that experience occurs during sleep, the second claim, that dream reports accurately represent dream content, is a more plausible target. A further analysis of the accuracy of dream reports is justified. If we reject this second claim, we can overcome the inconsistencies of the received view whilst being consistent with current scientific data. I propose a “narrative fabrication” thesis of dreams. According to this view, dream reports are often confabulated so that the narrative reported is not accurate of the dream experience itself. The main reasons this occurs, which I discuss in Sections “Rationalization of Strange Content” and “Confabulation and Memory Loss,” are that dreams can be bizarre which leads to rationalization of their content, and memory of dreams is poor, which can cause confabulation of dream events. Certain reporting conditions such as attaining lucidity can help to minimize confabulation, which explains why dream reports can at times correlate with dream behavior. However, the frequency of confabulation or fabrication cannot be entirely known using current methods. I begin by explaining how the narrative fabrication thesis can solve some of the problems of the received view.

### A BETTER EXPLANATION FOR SUSPENSE DREAMS AND INCONSISTENT CORRELATIONS

Firstly, the narrative fabrication thesis provides a more convincing explanation as to why eye scanning does not consistently correlate with non-lucid dreams reports, but is more consistently correlated with lucid dreams. If dream reports were largely fabricated by the waking mind and we rely on the accuracy of the dream report to make correlations, then it would be very difficult to consistently correlate dreams with behavior. We have reason to suspect that non-lucid dream reports are more prone to narrative fabrication than lucid reports. This, as I discuss in more detail in the following section, is because cognitive abilities such as memory and rational capacity are better retained in lucid dreams (Kahan and LaBerge, 1994; LaBerge, 2000; LaBerge and DeGracia, 2000). This would explain why research of lucid dreams has consistently been able to correlate eye movements and muscle twitches (Schatzman et al., 1988), because lucid dream reports are generally more accurate than non-lucid reports. Under the received view of dreams, this difference between lucid and non-lucid dream correlations is not convincingly explained. Narrative fabrication also would explain why sleep talk and other sleep behaviors have such tenuous connection with dream reports. The dream reports are often fabricated by the waking mind, and when the reports are fabricated, they do not correlate with sleep behaviors.

Secondly, the narrative fabrication thesis provides a plausible explanation for suspense dreams. The suspense element of suspense dreams is best explained by fabrication or confabulation of the dream report after waking. If dream reports are highly fabricated or confabulated, the “suspense” element might be part of the confabulation. Rather than “predicting” the external stimulus, elements such as the suspense, the feeling of “building up” to an event and even the apparent “seamlessness” in which the stimulus is incorporated into the narrative are confabulated elements of the report narrative that do not accurately describe the original dream experience. For example, in Dennett’s opera singer example, the chronology of the events in the report may not be accurate of the actual dream, and the suspense and narrative structure may be added in the report. Perhaps there was no water involved in the dream until the dreamer bent over, and the water falling on the soprano prior to that was a confabulation. The saloon scene in the other dream example might not have preceded the shooting in the way the report claims. Rather, the shooting may not have fit into the narrative seamlessly at all, but instead the waking mind confabulates such elements so that the narrative is more logical or more like a film narrative where events are foreshadowed.

Confabulation of the report narrative after waking is far more plausible than the cassette view. In the following, I discuss reasons that confabulation or fabrication can occur. Firstly, I compare confabulation in dream reporting with waking confabulations and set out how the two differ. Then I argue that bizarre and unusual experiences are prone to confabulation and rationalization. In other words, we have a tendency to make unusual or bizarre stories more coherent. Subsequently I discuss ways in which poor memory of dream content can lead to confabulation.

### DREAM NARRATIVE FABRICATION vs. WAKING CONFABULATIONS

People confabulate when they report sincerely and with conviction false beliefs about the past or the present or false explanations about their current attitudes and behaviors. Confabulation can occur in the presence of dementia, amnesia, or delusions for instance, but is also common in normal cognition (Bortolotti et al., 2012, p. 102).

In this section I draw on the literature of waking confabulations to compare with fabrication of dream reports. I note that dream reporting shares common elements with confabulation, but can involve other non-confabulatory elements which add to the inaccuracy of dream reports. Hence I use the more broad term “fabrication” as opposed to “confabulation.”

Early work on confabulation by Bonhoeffer (1901) and later confirmed by Berlyne (1972) distinguished two situations when confabulation occurred in patients with Korsakov syndrome, a disorder in which patients with severe memory loss confabulate past events. Bonhoeffer notes that patients often confabulate due to embarrassment caused by memory blanks, and other situations in which a patient goes beyond the needs of hiding the impairment, creating fantastic or adventurous scenarios. He notes a link between these stories and a delirious, dream-like state. Confabulations in waking patients are often linked to dream like states such as daydreams Scheid, 1934; Whitty and Lewin, 1961). I later discuss why such states are often linked with report fabrication, because of their bizarreness and poor memory retention. Berlyne (1972, p. 38) defines confabulation as “a falsification of memory occurring in clear consciousness in association with an organically derived amnesia.” This definition excludes falsification of memory due to delirious states. Dreams have been described by some as a form of delirium (Hobson, 1988; Hobson et al., 2000). If dream narrative fabrication were related to delirious aspects of dreams, then Berlyne’s (1972) description would exclude dream report fabrication. When I discuss confabulation in regards to inaccuracy of dream reports, I do not mean to exclude the possibility that dreams can be delirious, so a more appropriate definition is that of Metcalf et al. (2010, p. 65) who state that confabulations are “statements or actions that involve distortions or misinterpretations of memories without the conscious intention to deceive.”

Bortolotti et al. (2012) note that confabulations are usually formed when sufficient evidential support for a belief is absent, and are resistant to evidence that disproves the belief. Similarly, in a set of experiments on mild to sever confabulators, Shapiro et al. (1981) found that confabulators have difficulty monitoring the accuracy of their performance, and have difficulty using cues to aid performance. In dream reporting, there is no external evidence that can provide cues or support for or against a particular dream report, other than inconsistency between earlier and later reports of the same dream. Shapiro and colleagues refer to cognitive deficits in confabulators, whereas the dream reporter, who is most likely cognitively unimpaired, is reporting a previous state in which they may have been cognitively impaired. Unlike in most cases of confabulation, the dream reporter is not typically in a delusional state (when reporting)^11^. However, since some dreams may themselves involve delusional elements^12^, these elements may increase the dreamer’s propensity to confabulate upon waking. This might lead to difficulty reporting accurately.

I use the term “fabrication” instead of “confabulation” as firstly, I do not believe that all of the inaccuracies in dream reports can be confirmed to be *sincere* false beliefs, and secondly, people who report their dreams are not necessarily convinced that their own report is accurate. Regarding the former, some people may be motivated to distort or leave out embarrassing elements of dreams. This differs from Bonhoeffer (1901) discovery that confabulation can be cause by embarrassment, because for confabulators the embarrassment is caused by having poor memory. In contrast, the dreamer knowingly distorts the report due to embarrassing content in their memory. Regarding whether dream reporters are convinced by their reports, I think dreamers are often unsure and admit to having poor memory. In a dream report from Arkin et al.’s (1966, p. 205) research that I discuss in more detail in Section “Confabulation and Memory Loss,” the dreamer reports “Uh-uhm-uh-gee I- something about- yeah- a hat[…]” which indicates that the dreamer admits his memory of the dream is poor, but is trying to work out the details of the memory. So I refer to narrative “fabrication” instead of “confabulation” so as not to exclude other elements that can cause inaccuracy, although confabulation is certainly one of these elements.

I conclude that some of the explanations and mechanisms of confabulation in delusional patients may be relevant to dream reporting. Conversely it is clear that dream reporting is not entirely analogous with waking confabulation, and there are other elements involved in narrative fabrication. Commonalities include memory recall deficits, the possibility of reporting fantastical events, lack of ability to reality check and lack of evidential support for beliefs. Differences include the fact that dream reports can be fantastical but plausible, i.e., the dream itself can be fantastical, whereas reports of waking events cannot. While dreaming we undergo cognitive deficits, but not during the reporting of the dream, whereas the delusional confabulator suffers from deficits *while* making the report. This explains the waking confabulator’s inability to fact check and inability to support their view with plausible evidence. In contrast, the dreamer lacks the ability to fact check and gain evidential support not because of a cognitive deficit but rather because dreams are not externally verifiable and we cannot provide evidence to support their plausibility. Additionally, dreams can be fantastical, so it is not possible to test for plausibility. Dream reports can involve confabulation, but they are in important ways different from the confabulated reports of waking events made by cognitively impaired patients. What remains is to provide evidence for the fabrication of dream reports and discuss to what extent are dream reports fabricated.

### RATIONALIZATION OF STRANGE CONTENT

In this section I argue that dream narratives often are confabulated upon waking because of the nonsensical content of dream experiences. I assume Montangero’s (2012, p. 170) broad definition of “narrative” as “any report of a sequence of events involving persons or animals who react to one another or to physical events.” I agree with Montangero’s assessment that dream reports are generally more comparable to informal waking reports rather than stories, although dream reports may involve elements of story-telling, when a person reporting their dream is motivated to alter the narrative, for example, due to embarrassing content. These elements, however, could be controlled for by making reports anonymous. Instead I focus on the fabricated elements that are more akin to waking confabulations, as set out in the previous section. Montangero argues that “dreaming cannot be considered as the production of disorganized mental content since dreams have narrative features” (p. 169). In contrast, I argue that many of these narrative features may be a production of the waking mind rather than the dreaming mind. The waking mind often reconstructs the experience into a more coherent narrative, and this may happen as we try to remember and report the dream. I discuss studies which show that waking reports are also rationalized over time and argue that, given current technology, we cannot know to what extent a dream has been rationalized even if the dreamer is woken directly from REM sleep. There are no failsafe conditions to guarantee that a dream report will be accurate, although certain conditions will increase the probability of an accurate report.

Owen Flanagan considers the possibility that dreams are “simply noise left over from the work a mind designed for a day job continues to make on the night shift” (Flanagan, 2001, p. 40). This noise is interpreted into narratives by the conscious sleeping mind. Foulkes (1982, 1985) similarly describes dreams as the sleeping conscious mind’s attempt to organize unconscious activation of memory units. In this view, the dreaming mind does a good job of composing realistic narratives, which explains why lab-based reports are only occasionally unrealistic and contain bizarre features^13^. My view in contrast is that although experience occurs during sleep, the narrative of the dream is altered or fabricated by the waking mind.

The narrative of a dream may be composed partially or entirely upon waking^14^, but there is evidence supporting that dreams themselves have some narrative structure. If dreams were only disordered phenomenal sensations without narrative, all dream reports should be similar to what Hobson (2002) refers to as “internal percepts,” simple, unimodal sensations that lack narrative. The sensation of falling that many experience when falling asleep is an example of this. However, even bizarre dream reports are distinct internal percept reports in that they have some narrative structure, involving characters and events occurring over time. Some evidence indicates that dreams are convincing simulations of waking life and waking concerns (Snyder et al., 1968; Snyder, 1970, p. 127; Domhoff, 2007). I argue that it is likely that bizarre dream content is often rationalized by the waking mind.

Foulkes (1979, 1999) observed that several forms of selectivity occur in dream reporting when the dreamer has time to confabulate. Foulkes (1979) compared REM and non-REM (NREM) lab awakenings with natural morning awakenings in the same subject, and found that in the morning reports, there is a tendency to rationalize dreams, as subjects assume that they would make rational decisions and that outcomes would be rational. Foulkes notes that there can be, during dreaming, a remarkable dissociation, by waking standards, of feeling and action. One commits atrocities, with no remorse. One stands naked, with no guilt or shame. What may happen in [morning] recall is that waking rationalization supervenes and imposes feelings on events wherein none were in fact experienced: “Because I did this, or because this happened to me, I must have felt this” (Foulkes, 1979, p. 246).

In one REM awakening, a subject initially reported a dream of being chased but feeling no fear during the dream. In the later morning report of the same dream they described being afraid (Foulkes, 1999, p. 26). Foulkes suggests that the waking mind of the subject rationalized that the dream should have been scary, so misremembered it thus. Dreams lack appropriate *binding* of multiple sensory modalities (Revonsuo, 1995), i.e., the subconscious process of joining together multiple aspects into a single, coherent experience. The waking mind may rationalize these mismatching modalities to form a more coherent narrative.

Evidence for the rationalization of ordinary waking memory content is demonstrated by Bartlett’s (1932) work, in which subjects were asked to remember a story called “The War of the Ghosts.” The story involved supernatural elements such as ghosts and spirits. Bartlett notes that when asked to recall the story, many subjects left out most or all supernatural occurrences, despite the salience of these events to the narrative. These elements were replaced with more familiar cultural symbols or behaviors that were not in the original story, such as a dying man’s face turning white. Alba and Hasher (1983) describe this as a schematic process in which the memory is selected, abstracted, interpreted and integrated. They describe these four forms of construction in memory thus:

selection – a process that chooses only some of all incoming stimuli for representation; abstraction – a process that stores the meaning of a message without reference to the original syntactic and lexical content; interpretation – a process by which relevant prior knowledge is generated to aid comprehension; and integration – a process by which a single, holistic memory representation is formed from the products of the previous three operations (p. 203).

Therefore, rationalization of unusual elements occurs in waking narratives. If dream narratives involve many bizarre elements, rationalization can occur, although since we cannot compare the report with the original dream experience, it is unclear to what extent.

This analogy between the war of the ghosts and dreaming is not perfect, since months passed between the reading of the story and the report, whereas dreams are usually reported directly after waking. However, as I discuss in the following section, memory loss for dreaming is generally far more rapid and dreams are more bizarre than waking memories, so the reconstruction process may also occur more rapidly, perhaps immediately upon waking or during the reporting of the dream. Poor memory during sleep may exacerbate confabulation, as it does in patients with confabulation disorders.

### CONFABULATION AND MEMORY LOSS

Memory loss is an important complicating factor regarding dream report collection. Upon waking, memory of dreams is highly reduced and such memory deficits can lead to confabulation of reports. Many sleep experiences fade completely upon waking. Frequently, one is left with very poor or no recollection. For most dreams to be remembered, mental effort is required. Upon waking, we attempt to retain residual impressions of the experience, and during that process we assemble impressions into a coherent narrative. Training and practicing remembering dreams and making dream reports may improve reporting accuracy, but it is not clear to what extent. Schredl et al. (1996) and Schonbar (1965) note that if subjects are interested in dream reporting, have a positive attitude towards dreams or are particularly well motivated, dream reporting is often more frequent. However, increased dream reporting does not entail that these reports are accurate. For example, in Arkin et al. (1966) attempt to improve dream recall and cause sleep talk to verify reports by hypnotizing subjects before sleep, even these reports are often pieced together as the dreamer attempts to remember the specifics of the experience. For example:

Uh-uhm-uh-gee I- something about- yeah- a hat- belonging to you know- one of those old sheep herder hats- I don’t remember what it was all about though.

This report was correlated with sleep speech:

Mm- mmm- how about this hat (?) from India where- where it was made by camel driver- damn thing [pause] pretty little bugger. (The (?) indicates a word which was unclear) (Arkin et al., 1966, p. 205).

This report was made directly after an REM sleep awakening, nonetheless the details are very vague, and much of the dream forgotten. The sleep talk differs from the report, which does not mention a camel driver, India, or that the hat was pretty. There is inconsistency between the sleep talk referring to a shepherd, and the report about a camel driver. It is unclear whether the sleep talk is about a hat at all, since the researchers assumed that an unintelligible word was “hat.” This demonstrates that even direct REM sleep awakening reports are subject to rapid memory loss. Although waking directly from a dream most likely increases the accuracy of the report since there is less time to confabulate, an accurate report is not guaranteed.

Reporting and rehearsing a memory can affect what is remembered. Rehearsing causes certain salient parts of the event to be remembered in detail whilst other parts of the event are forgotten. In a study of 56 participants by Horton (2011), the test group rehearsed their dream reports, and the control group did not rehearse. It was found that rehearsal did not increase the richness of detail of memory, but rather caused the dream to be remembered in precisely the same way as it was initially reported. The group that did not rehearse the dream reported different elements of the dream at different times. This suggests that when a report is rehearsed, the report is remembered as opposed to the dream itself. Horton concludes that “the effect of rehearsal can limit what is later recalled, as well as enhance it. What is recalled is the report, rather than the original dream or event itself” (Horton, 2011, p. 12). It is unclear whether rehearsal enhances some memories or rather leads to fabricated elements being remembered instead of the dream itself.

All memory is subject to forgetting, however, I argue that dream memory is distinctively problematic. Memory is not like a notebook in which information is stored for later retrieval; rather it is complex and involves multiple events entwining, often with forgotten items filled in by unrelated events and confabulation. Sutton (1998, p. 2) describes memories as being “blended, not laid down independently once and for all, and […] reconstructed rather than reproduced.” Memory of an event may be reconstructed with elements of many separate events. When details of an event are forgotten, this can lead to “filling in the blanks” and blending memories together. For Schechtman (1994), this blending, summarizing, condensing, and conflating of memories is necessary for our sense of self. She argues that “it is precisely insofar as our memories smooth over the boundaries between the different moments of our lives, interpreting and reinterpreting individual experiences in the context of the whole, that we are able to produce a coherent life history” (p. 13). This describes what many would refer to as the failings of memory as opposed to essential processes that enables us to see our memories as part of an integrated whole. Sutton (2003) notes that reconstruction does not necessarily render memories *false, *but rather reconstruction is a necessary condition for remembering since all memories are reconstructed. Remembering isn’t replaying the event in one’s mind exactly the way it was, but rather interpreting and integrating memory traces. Reconstructed memory can be veridical but different from the initial experience, for example when the observer switches perspectives, viewing the event from an angle which they did not perceive initially (Rice and Rubin, 2009), e.g., a bird’s eye view, known as a switch from “field” to “observer” perspective. The sequence of events remembered may be accurate despite the original perspective not being represented accurately. Reinterpretation, argues Sutton, is the norm, not the exception. One might argue from this that reinterpreted dreams aren’t necessarily false memories, however, dreams are especially problematic for a variety of reasons.

If a memory can be accurate even if the perspective has been switch from field to observer, it is unclear what to make of the switching of perspective in dreams^15^. A dream report in which the observer is looking down from an observer or birds eye perspective is usually interpreted as if the observer *in the dream *was in fact looking down from above. In contrast, in a waking report we can infer that during the event, the observer did not literally switch perspectives, as that would be physically impossible. So it is not clear what to make of the veracity of such a report. We do not know whether the dreamer was literally looking down from above or rather the perspective shift occurred only in memory.

A further issue is that, even if memories can shift and blend and still be “accurate,” many memories are not accurate, and we need to determine the accuracy of dream memories. Dreams fade much more quickly and are more difficult to retain than memories of waking events. Most dreams are forgotten instantly, especially if we do not wake immediately after dreaming. Hobson (2005) remarks, memory for dreams in subsequent waking is notoriously poor. […] I am a relatively good dream recaller, and I consistently record those that I remember. My collection of reports is on the order of 400 entries in a journal that I have kept for 30 years. That is just a bit more than 1 a month! […] If I were that amnestic in waking, I would be in a mental hospital (p. 27).

In contrast, Beaulieu-Prevost and Zadra (2005) argue that well motivated dream aficionados can report dreams quite regularly, some report dreams every morning. Nonetheless, even the most motivated dream reporters forget most dreams. Multiple sessions of REM sleep occur every night and dreaming occurs in most of these sessions and 80–90% of REM sleep awakenings lead to reports (Domhoff, 2003, p. 17), but individuals rarely report more than one dream upon waking in the morning. Therefore even regular reporters forget most dreams. Reduced memory capacity can lead to greater confabulation when attempting to recall events, so we should expect less accuracy in dream reports.

Another difference between dream and waking memory reports is that other observers can verify waking memories. Waking memory can be easily discredited when stories conflict with external evidence. In contrast, the dream environment is internally generated, so as Malcolm argues, the dream content cannot be externally verified. We cannot use cues from the external environment to assist with memory of dreams, and dream memories cannot be evaluated for plausibility since dreams can be bizarre and fantastical. In the following section I will discuss a final problem afflicting dream reporting, which is that some dreams involve altered states of consciousness that cannot be reported accurately.

### ALTERED STATES OF CONSCIOUSNESS IN DREAMS AND THEIR COMPREHENSIBILITY

One possible explanation for unreliability of dream reporting is the change in brain function that occurs while dreaming. During REM sleep, the brain generally exhibits increased activation in sensorimotor areas, visual areas and emotional centers, whilst there is decreased activation in the dorsolateral prefrontal cortex (DLPFC) and memory storage and access areas (Hobson et al., 2000). With an altered neuromodulatory system and changes in activation in the brain, we may lack some abilities associated with normal functioning consciousness^16^. The experience that occurs in this altered state of consciousness might be difficult for the waking mind to comprehend. Significant alteration in mental state may mean that the experiences occurring in that state are incomprehensible to the normal waking mind, just as a bizarre hallucination might be difficult to comprehend, remember and describe. If a dream experience is sufficiently different from waking experience, the waking mind might need to confabulate to make the experience reportable. Perhaps a fully functioning waking mind cannot comprehend the altered state. The waking mind may try to rationalize the experience so it can be reported in a meaningful way, a process that goes far deeper than a few rationalized bizarre elements; rather the entire narrative is a fabrication.

Some evidence suggests that there is a carry-over state after waking in which certain attributes of the brain in REM sleep persist. Most have experienced this state directly after waking; confusion, disorientation and failure to realize they were just dreaming (Balkin et al., 1999; Reinsel and Antrobus, 1992). Some argue that dreams that are reported directly after waking from REM sleep can be highly accurate since the carry-over effect allows for the mind to remain in an altered state. This may also explain why fewer reports are elicited in the morning compared with REM awakenings. However, it is unclear whether this waking stage retains the altered state of REM sleep, or whether it is an in-between state. A carryover state would not necessarily elicit clear and coherent dream reports, since an individual who has just woken and is confused and disoriented would most likely report inaccurately. This is strongly suggested by the Arkin et al. (1966) example discussed in Section “Confabulation and Memory Loss.” The participant mumbles in a barely coherent way and the narrative remains quite unclear.

The issue of confabulation of dream reports is complicated by the possibilities that certain types of experience are more likely to be confabulated than others, and that certain individuals are more susceptible to fabrication. I argue that it is difficult to determine whether fabrication occurs and to what extent.

## INDIVIDUAL DIFFERENCES IN DREAM REPORT FABRICATION

In this section I argue that certain individuals are more prone to confabulation than others, but dreams are more prone to confabulation in general than waking reports. Research indicating individual difference in dream recall (Schonbar, 1965; Schredl et al., 1996), a relation between dream recall and interest in dreams (Beaulieu-Prevost and Zadra, 2005) and relationships between dream recall and gender (Schredl, 2002–2003) suggest that there are many differences between individuals regarding the number of dreams that are recalled and reported in the morning. This might suggest there are also individual differences regarding the accuracy of dream reports. However, this claim is much harder to test, as report frequency can be determined simply by keeping track of the number of reports. Determining report accuracy requires comparison between report and dream, which is not currently possible.

Garry et al. (1996) demonstrate that if someone *imagines *an event, they are more likely to later believe that it occurred than events they had not imagined. They coined the term *imagination inflation* to refer to the tendency in adult subjects to judge childhood events that they had imagined as more likely to have occurred than events they did not imagine. Imagination inflation also occurs when subjects imagine *future* events, and judge whether the event is likely to occur (Carroll, 1978; Gregory et al., 1982). This is consistent with the theory that remembering is *goal driven* as opposed to solely accuracy driven (Conway, 2005; Sutton et al., 2010). According to this view, memory involves principles of both *correspondence*: accurate representation of real events, and *coherence*: the maintenance of narrative coherence of events over time that make sense to the individual. There are multiple functions of memory, of which accurate portrayal of past events is only one (Boyer and Wertsch, 2009). Imagination might similarly affect dreams; if someone imagines dreaming the event, they are more likely to later believe they *have *dreamt it. Although it has not been tested, dreams may be even more prone to imagination inflation since dream events are often difficult to distinguish from imagined events. Here I argue that dreaming is subject to a higher degree of imagination inflation due to poor memory, bizarreness of dreams, and because we are unable to assess the likelihood that a dream even occurred by judging its plausibility.

Heaps and Nash (1999) discovered that there are individual differences in imagination inflation. They explain that while hypnosis is sometimes used for recovering lost memories, certain individuals are prone to report false memories under hypnosis due to suggestions from the hypnotizer. A famous example is the case of Paul Ingram, a man who began to report bizarre false memories about incest and Satanist cults after hypnotic suggestion (Ofshe, 1992). This gives further reason to be wary of aforementioned experiments of Arkin et al. (1966, 1970a,b), which involved hypnotism. Heaps and Nash used the Gudjonsson Suggestibility Scale (GSS; Gudjonsson, 1984) to identify interrogative suggestibility (susceptibility to the influence of an authoritative questioning source) and hypnotic suggestibility (susceptibility to influence under hypnosis) in test subjects. They found that individuals who are prone to hypnotic suggestion are also more prone to imagination inflation, i.e., to mistake imagination for memory. This is linked with *dissociativity*, defined as failure to “distinguish and integrate memories, fantasies, motivations, and actions in awareness (Spiegel, 1995, Whalen and Nash, 1996)” (Heaps and Nash, 1999, p. 314). Dissociative individuals experience disrupted memory, confuse real events with fantasy and are more likely to mistake imagination for memory.

Heaps and Nash (1999) interviewed subjects on how likely it was that they had experienced certain events before the age of 10, such as whether they had broken a window with their hand. 2 weeks later they were asked to perform the same interview again under the pretence that the initial responses had been lost. They found that people who are dissociative or prone to hypnotic suggestion were more likely to report in the second interview that they *had *broken a window with their hand despite initially denying remembering the experience. Heaps and Nash suggest that because dissociative individuals are unable to distinguish and integrate memories and fantasies, they rely less on their memories and more on inferences and external evidence. Normal, waking individuals also demonstrate such reliance, but to a lesser extent. French and Richards (1993) found that when asked to draw a Roman numeral clock, almost all participants drew the 4 in the clock as “IV” despite the fact that Roman numeral clocks represent the 4 as “IIII.” They explain that individuals rely on their schematic knowledge of the way numerals are represented rather than their memory of the clock. This commonly occurs in cases where normal participants cannot rely on their memories. However, dissociative patients, who suffer from frequent disruptions of episodic memory are forced to rely more heavily on schematic knowledge, and have lower standards for accepting memories as real (Heaps and Nash, 1999, p. 317).

Individuals who are prone to hypnotic suggestion or dissociation and who find it harder to distinguish between autobiographical memories of experienced events and semantic memories or imagination may be less able to distinguish between dreams and other memories. For example, they may find it difficult to distinguish between fantasizing before falling asleep and the content of their dreams. Screening research samples for those who are more likely to confabulate might be necessary for accurate dream research, although a separate experiment may be required to ascertain whether the same individuals are more prone to confabulate dream reports. I propose that a similar experiment to Heaps and Nash’s (1999) could determine the extent of imagination inflation differences in dreaming. Firstly, participants would be asked to imagine a specific event before falling asleep, and then researchers would compare whether a dream report the following morning or during an REM awakening involved the imagined elements. Differences between dissociative and normal participants could then be ascertained^17^. I predict that dreaming is highly susceptible to imagination inflation in all individuals: in many dreams it is difficult to distinguish and integrate memories, fantasies, motivations and actions in awareness due to decreased memory and other cognitive capacities. If this prediction is correct, there would be less difference between dissociative patients and normal controls regarding the amount of imagined events incorporated into the dream report than we see in waking memory reports.

Heaps and Nash’s (1999) description of “dissociativity” as disruptions in episodic memory leading to difficulties distinguishing between real and imagined events is an accurate description of many dream states. Barrett (1995) argues that many of the cognitive features of dreaming are similar to those of dissociative states. This is supported by experiments carried out by Johnson et al. (1984) who found that subjects generally have difficulty discriminating between their own dreams and reports of others’ dreams. They argue that this is not simply an issue of poor memory, but that dreams are “deficient in conscious cognitive operations that help identify the origin of information generated in a waking state” (p. 329). Since dreams are generated unconsciously, they often lack important cues that assist in distinguishing imagined from experienced events.

Another reason to suspect that dreams are often hard to distinguish from other mental states is that we cannot “reality-test” to distinguish between imagination and dream memories. Waking memory can be judged as plausible if it is consistent with other waking events. This is not so for dream memories, since dreams need not be plausible by waking standards. Since neither dreams nor imaginings need to be plausible events, we cannot reality-test to distinguish whether I dreamed or imagined something.

I have highlighted some reasons why dream reports are more prone to fabrication than waking memories, and proposed an experiment to test whether dream reports are prone to imagination inflation. In the following section I will discuss what evidence could verify or disprove the narrative fabrication thesis, or rather how we could discern to what extent fabrication occurs.

## VERIFYING FABRICATION

Dreamers report a range of experiences anywhere from highly bizarre and incoherent to realistic simulations of waking events. I have suggested that highly incoherent dreams are less likely to be reported accurately, since bizarre elements get rationalized, but does this mean that bizarre dream reports involve a weaker degree of fabrication? How can we report bizarre dreams at all?^18^ To the former I would respond that bizarre reports are not necessarily indications of lesser fabrication, since the initial dream experience may have been even *more* bizarre. It is unclear what extent rationalization occurs. In the case in the war of the ghosts, not all participants left out the supernatural elements, so individual differences in narrative rationalization may occur in dream reports as well. We still report bizarre dreams (as opposed to only reporting normal, waking life-like dreams) because rationalization is not consistent across reports, and levels of bizarreness most likely alter in dreams. We sometimes report bizarre waking experiences and bizarre, supernatural stories, however, sometimes we rationalize away the bizarre elements. My contention is that it is unclear how much the narrative has been altered by the time the dream is reported. As with the war of the ghosts experiment, bizarre narratives are often rationalized into more coherent narratives but not *always* or to the same extent. Unlike the war of the ghosts experiment, the report cannot be compared with the original experience and confirmed. The mind may not be able to rationalize away *all *bizarre features into a completely coherent narrative, which is why bizarre dreams are still reported. Some may argue that current experiments on lucid dreaming and REM sleep behavior disorder provide verification for dream content, however, I disagree. I will argue that better methods are required to determine to what extent dreams are fabricated. I will then suggest some methods that could be carried out in the future.

Lucid dreaming tasks in which a lucid dreamer performs activities such as drawing letters, and signaling when the activity is complete may be a method of increasing the accuracy and providing some verification of dream content as well as providing strong evidence that the dreamer is conscious and aware they are dreaming. However, such an experiment would not failsafe against all report fabrication. It may confirm the dreamer is performing a specific activity, yet it would not verify other dream elements. For example, a strange setting may still be rationalized away or forgotten in the report because of its unusual nature. Thus, even lucid dream reports under strict conditions may involve confabulation. A second issue is that lucid dreams are not representative of all dreams. Researchers who focus on non-lucid dreams cannot assume that they are similar to lucid dreams in all relevant aspects. I suspect that lucid dream reports are in general more accurate than non-lucid dream reports due to increased memory and rational capacities. Later in this section I will propose a method to test this. However, lucid dreams cannot replace non-lucid dreams in research for the purpose of minimizing report confabulation because they are not representative.

Rapid eye movement sleep behavior disorder as discussed in Section “Sleep as Defined by Neuropsychology and Lucid Dreaming Confirm that Experience Occurs During Sleep” provides another example in which dreams can be corroborated with sleep behavior. However, it is important to note that RBD is caused by neurological disorders which can also affect the content of dreams (Schenck et al., 1987). For example, RBD sufferers experience a higher frequency of violence in dreams, so should not be seen as indicative of all dreaming. Secondly, although RBD gives some evidence that experience is occurring during sleep, such behavior only provides vague evidence of general dream content. Schenck et al. (1987) note a case in which a sleeping man who was throwing punches woke up to report that he was having a dream of fighting a squirrel in the attic. Mahowald et al. (2005) report a case of a man who killed his girlfriend while dreaming of fending off an intruder. Although violent behavior can be correlated with reports of violent dreams, the behavior does not inform an outside observer of the specifics of the dream content. We cannot judge from behavior alone that the first man was attacking squirrels in his dream, or that the second was fending off an intruder. Also, it is unclear if the reported dream correlated with that particular session of behavior, or if it was a previous dream. Perhaps the reported dream and the behavior were not from the same session. So RBD behavior does not verify dream content, and does not safeguard against narrative fabrication.

Some theorists may find such skepticism unsatisfying, as our current methods do not verify specific dream content or and cannot confirm or deny predictions regarding the extent to which fabrication occurs. However, with improved technology and improved methods, this may not always be the case. As aforementioned, we cannot currently verify cognition from neuroimaging, however, progress in our understanding of the brain and advanced technology may one day make this possible. Coltheart (2006) argues that neuroimaging is uninformative because we would need a full explanation of brain function for brain images to be useful, but once our understanding is complete we would no longer need images of the brain to understand the brain. However, it is not quite true that such technology would be uninformative for other purposes. We could use real-time function brain activity images to discern the cognitive state of or receive communication from an individual that was otherwise unable to communicate, for example, patients in comas, locked in syndrome, or dreaming subjects. So although I agree with Coltheart’s assessment that with current technology we cannot discern cognitive states from brain imaging alone, in the future this may be possible. With this technology we could ascertain accurate information about dream content. It is possible that in the future, neuroimaging or other technologies will be about to discern even what a person is dreaming while they sleep. Perhaps outside observers could experience someone else’s dream. One intriguing although somewhat unnerving possibility would be to could recreate a “film” of someone’s dream while it happens based on their neural activation. This information could then be compared with dream reports to ascertain to what extent we fabricate reports. However, this is a far off possibility, and I would like to propose an alternative that would be achievable with current technology.

An exciting possibility that does not require futuristic technology would be to extend research into sleep signaling in an attempt to have subject signal specific content of the dream with eye movements. Eye signaling experiments discussed above demonstrate that this is possible, however, these experiments not intended to verify dream content, but rather to demonstrate whether bodily movements correlate with dream content. With a greater focus on verifying dream content, this could be achieved. Schatzman et al. (1988) have demonstrated that a lucid dreamer eye signals do in fact correlate with dreaming eye movements, and dreamers can signal that they are dreaming. However, one of Schatzman’s experiments also provides evidence that it is possible to communicate more specific content of the lucid dream as well. Worsley, an expert lucid dreamer, carried out instructions to draw large numbers on the ground after lucidity onset. He first indicated lucidity by performing a set of prearranged eye movements. Then he drew the numbers while tracking his arm movements with his eyes. He signaled the completion of each number with eye flicks corresponding to the number drawn (one flick for number 1, two flicks for number 2 etc). All of these eye movements were recorded by EOG. The electromyography (EMG) strapped to his forearm picked up the tensing of his muscles which correlated with the writing movement. This raises the possibility of directly reporting dreams whilst dreaming. Although the eye flicks only reported the numbers that were being drawn by the dreamer, this can be seen as a direct communication of some of the content of the dream. However, since the aim of the experiment was to determine whether bodily movements correlate with dream body movements, further effort was not made to directly communicate other more specific dream content.

LaBerge (1994) notes that attempts were made to teach lucid dreamers to use sign language to convey dream content whilst asleep. This research paradigm used a sensor glove to attempt to record the movements. At the time of publication, existing technology was not sensitive enough to read the hand signals and to my knowledge, there have been no recent successful attempts to achieve this. Perhaps reduced muscle tone during sleep would make it unlikely for hand signals to be sufficiently strong for recording^19^. So it is unlikely that hand-sign language could be used to communicate dream content. However, as previously mentioned, eye movements have proven to be successful thus far in indicating a few elements of dream content. I propose that a type of eye movement sign language could be adopted to verify more detailed dream content, and communicate directly while dreaming. An example of eye movement communication can be seen in the case of patients with locked in syndrome, a disorder in which patients are completely paralyzed except for their eyes (Chapman, 1991). The primary method of communication for people with locked in and other similar disabilities is “eye typing” (Majaranta and Raiha, 2002). This method uses a virtual keyboard and eye tracker so that the individual can type words by directing their gaze at the desired letter on a keypad. This method of course could not be used for dreaming patients due to the input blockade as the dreamer could not look at a keypad whilst asleep. However, an alternative method of eye movement sign language could be devised. The method that springs to mind is Morse code. A signaling system using left and right eye flicks instead of long and short taps could be learnt by expert lucid dreamers and used to communicate dream content. For example, the word “dream,” which in Morse code is [-/-//-/- -] (where is a short tap, - is long, and / refers to a pause) would be indicated with left and right eye flicks as following: [RLL/LRL/L/LR/RR]. Methods of EOG signal processing can be used to detect eye movement directions, as shown by Merino et al. (2010). Yet the difficulty and complexity of such an experiment is obvious.

Firstly, learning such a complex sign language would be difficult and being able to replicate it during a dream would require participants to be both dream-code and lucid-control dreaming experts. Maintaining sufficient concentration during even a lucid dream is difficult. Secondly, the time it takes to signal even the word “dream” is significant, so there would be a limit on how much detail that could be reported. Majaranta and Raiha (2002) notes that practiced users of the eye-controlled virtual keyboard typing system can type about six words per minute. A quicker signal method could incorporate left, right, up, and down eye movements, so that the 20 most common letters would require only one or two eye flicks. This would speed up the process. Another problem is that there may be a disruption of the content of the dream when the subject intentionally disrupts their eye-movements. Tholey (1983) found that when lucid dreamers focus their eyes on a stationary object, it often causes them to wake up. It is unclear, however, whether controlled eye flicks would cause the same phenomenon. Signaling with eye flicks has been a method use both by LaBerge and colleagues, Schatzman and colleagues and other research groups as previously mentioned. They have not reported that eye flicking leads to shorter periods of dreaming, premature awakening or disrupted content.

The main drawback of this technique is that it can only be applied to lucid dreams. A dreaming participant would not be able to signal if they did not know they were dreaming. This is problematic because, as argued earlier, lucid dreaming is not representative of all types of dreaming. The increased metacognitive and other cognitive abilities including control, memory, and rational capacity mean that lucid dreams are probably less prone to confabulation than the non-lucid variety. So correlating dream reports with eye signaling reports would not verify the accuracy of dream reports in general. Such signaling reports would give strong evidence for the accuracy of lucid dream reports (if my prediction that lucid dream reports are more accurate than non-lucid reports is correct), but this could neither confirm nor deny the narrative fabrication thesis for non-lucid dreams.

The aforementioned future technologies and experimental methods would not only give us direct access to dream content, they would go some way towards demonstrating the extent to which report fabrication occurs between dreaming and the initial REM awakening report. Individual differences in report fabrication could also be researched. So although the narrative fabrication thesis is currently difficult to verify, this may be possible in the future.

## CONCLUSION

Malcolm’s verificationist criteria for dreaming are in many ways implausible given the current scientific evidence. I have argued that Malcolm’s and Dennett’s anti-experience views should be rejected. There is strong evidence that experiences occur during sleep. However, I have argued against the assumption that dream reports are generally accurate. Dream reports are not as reliable evidence for dream experience as waking reports are for waking experience. It is implausible to deny outright that experiences *do* occur during sleep but dream reports are often confabulated because of poor memory, confusion of bizarre dream content and changes in cognition. In these cases, dreams are difficult to remember and report, so confabulation and inference-making occurs.

It is well documented that narratives can be confabulated between initial REM sleep awakenings and subsequent reports. I argue that we have reason to suspect that fabrication occurs in the original report as well. Evidence for this is the tendency to rationalize strange elements in waking reports. Subjects tend to leave out supernatural or bizarre elements when reporting waking memories of stories, as exemplified in Bartlett’s war of the ghosts. Confabulation is exacerbated in dreams by rapid memory loss and bizarre dream content. Waking memory is a process of reconstruction and blending of elements, but unlike waking memory, we cannot reality-test for dream memories. Dream experiences involve imaginative elements, and dream content cannot be verified with external evidence. In summary, dream reports suffer from diminished memory, bizarre content, source confusion, and cannot be verified with external evidence. I have argued that although lucid dreaming provides verification of conscious experience during sleep, we cannot verify the specific content of dreams, nor rule out with certainty that a report has been fabricated.

I have proposed three methods to test my views. Firstly, I proposed a comparison between dissociative and non-dissociative individuals in imagination inflation of dream reporting. My theory predicts that there should be less difference between dissociative and non-dissociative individuals in dream reporting than in waking memory reporting because dreams themselves are dissociative. Secondly, I noted that in the future, new neuroimaging technology and methods might be used to get direct information about the content of dreams, and this would either verify or disprove my theory by comparing the direct content with the dream report. Finally, I proposed a new method of dream signaling. Instead of using hand-sign language as LaBerge attempted, we should incorporate eye-flick sign language. This method is limited in that it would only verify the content of lucid dreams, not non-lucid dreams, and I expect that lucid dreams would elicit more accurate reports than non-lucid dreams.

I do not argue that all dream reports are inaccurate or that all dreams are cognitively impoverished. Reports of dreams that involve higher cognitive functions, logical inferences and rationality are more likely to be reliable than cognitively deficient dreams. However, I have raised two main issues. Firstly, dream reports are generally much less accurate than waking reports, and secondly, we cannot use current scientific methods confirm the extent to which dream reports are confabulated. With the new methods that I have proposed and improved future technology, we may one day be able to discern the accuracy of dream reports and ensure accurate representation of dream content with external observer verification.

## Conflict of Interest Statement

The author declares that the research was conducted in the absence of any commercial or financial relationships that could be construed as a potential conflict of interest.

## Footnotes

1 Flanagan (2001); Foulkes (1999); Hobson (2002); Jouvet (1999); Metzinger (2009) and Revonsuo (1995) to name a few, support the received view.

2 Sosa (2005) similarly argues that anything that occurs within a dream occurs under an “in the dream” operator, which entails that the event, including thoughts, beliefs and other mental events did not *in fact* occur.

3 Dennett refers to these as “precognitive” dreams. However “precognitive” dreams also refer to a broader subset of dreams which appear to predict events but do not involve the same suspense aspect, or filtering in of external stimuli. E.g., I dream that my town gets hit by a cyclone, and two days later, it does. Since Dennett denies that these dreams could truly be precognitive, I find “suspense dream” to be a preferable term.

4 This view has in fact been rejected by most dream theorists. I will not review all of the arguments here, but note what I see as the weakest points of Malcolm’s argument, namely incorrect assumptions about being sound asleep. See also Ayer (1960); Brown (1957) and Dunlop (1977).

5 Extensive research has been done on lucid dreaming. See Kahan and LaBerge (1994); LaBerge (2000); LaBerge and DeGracia (2000); LaBerge and Levitan (2004); Spadafora and Hunt (1990). For discussion, see Windt and Metzinger (2007).

6 The received view does not imply the scanning hypothesis, but there would need to be an explanation why scanning only correlates with reports for some dreams.

7 Electrooculogram (EOG) measures sleeping eye movements, and Electroencephalogram (EEG) measures electrical brain activity.

8 To be discussed in more detail in the Section “Room for Skepticism: What the Received View Does Not Explain.”

9 Examples of this are discussed by Fine (2011), in which assumptions about the differences between male and female cognition lead researchers to search for evidence of small differences in brain activity. Whether such activity indicates differences in cognition is unknown.

10 In Section “Verifying Fabrication” I argue that this is the key to verifying dream content, so such a theory does not mean neuroimages no longer have anything interesting to contribute. Once all of the relevant data is known, then we *will *be able to use imaging techniques to discern specific content of dreams.

11 If we were to accept Dennett’s cassette theory, that dream reports do not describe sleeping experiences, we could consider the reporter to be in a delusional state since they would be reporting something that had not happened at all (thanks to a reviewer of this article for this interesting point). However I reject the anti-experience thesis, so I do not think that the dreamer is delusional when they are reporting the dream.

12 Hobson et al. (2000, p. 799) argues that “dreams are delusional; we are consistently duped into believing that we are awake unless we cultivate lucidity”. Although I do not agree that dreams are entirely delusional, I think we have good reason to concede that dreams can involve many delusional elements. For example, one might identify a character in a dream as their mother even though it looks nothing like their mother. A mundane dream about one’s mother in which she looks as she normally does, on the other hand, would not be delusional.

Foulkes (1996, 1999) and others argue that dreams are generally not as bizarre as we may believe, but rather home reports tend to be biased towards memorable, strange dream experiences. During lab awakenings, dreams reports often reflect normal, waking life.

14 This differs from Malcolm, who does not clarify when the composition of dream reports occur, and from Dennett, who suggests that the narrative is unconsciously composed by the mind during REM and we become conscious of the experience upon waking.

15 For a discussion of shifting perspectives in dreams, see Rosen and Sutton (2013) forthcoming in Philosophy Compass.

16 Not all dreams involve these neural and cognitive differences. For example Dresler et al. (2012) found lucid dreams involve higher cognitive capacities and has demonstrated increased neural activations in frontal and frontolateral regions of the brain. Also, lucid dreams demonstrate high levels of cognitive ability.

17 Since we cannot determine whether imagination is later incorporated into a dream (and hence a report of the imagination as a dream *would *be accurate) we cannot ascertain the accuracy of the report in the same way we could determine the accuracy of the waking report. However, if dissociative individuals report dreaming what they previously were imagining more often than non-dissociative individuals, the two possible explanations are that (1). Imagination inflation is occurring more often in dissociative individuals or (2). dissociative individuals are more likely to incorporate imagination into a dream. Given previous research on dissociative individuals, we would have more reason to support explanation 1.

18 Thanks to Thomas Metzinger for these points.

19 In their most recent paper, Kahan and LaBerge (2011) overview the strong evidence in support of lucid dreaming, including eye signaling, but make no mention of any progress using the hand signaling method.

## References

[B1] Alba J. W. Hasher L. (1983). Is memory schematic? *Psychol. Bull*. 93 203–231 10.1037/0033-2909.93.2.203

[B2] Arkin A. Hastey J. Reiser M. (1966). Post-hypnotically stimulated sleep talking. *J. Nerv. Ment. Dis.* 142 293–230 10.1097/00005053-196604000-00001 5946044

[B3] Arkin A. M. Toth M. F. Baker J. Hastey J. M. (1970a). The frequency of sleep-talking in the laboratory among chronic sleep-talkers and good dream recallers. *J. Nerv. Ment. Dis.* 151 369–374 10.1097/00005053-197012000-00002 4320643

[B4] Arkin A. M. Toth M. F. Baker J. Hastey J. M. (1970b). The degree of concordance between the content of sleep talking and mentation recalled in wakefulness. *J. Nerv. Ment. Dis.* 151 375–393 10.1097/00005053-197012000-00003 4320644

[B5] Ayer A. J. (1960). Professor malcolm on dreams. *J. Philos.* 57 517–535 10.2307/2023332

[B6] Balkin T. J. Braun A. R. Wesensten N. J. Varga M. Baldwin P. Carson R. E. (1999). Bidirectional changes in regional cerebral blood flow across the first 20 minutes of wakefulness. *Sleep Res. Online *2(Suppl. 1) 6

[B7] Barrett D. (1995). The dream character as a prototype for the multiple personality alter. *Dissociation* 8 1

[B8] Bartlett F. C. (1932). *Remembering: A Study in Experimental and Social Psychology*. Cambridge: Cambridge University Press

[B9] Beaulieu-Prevost D. Zadra A. (2005). Dream recall frequency and attitude towards dreams: a reinterpretation of the relation. *Pers. Individ. Dif.* 38 919–927 10.1016/j.paid.2004.06.017

[B10] Berlyne N. (1972). Confabulation. *Br. J. Psychiatry* 120 31–39 10.1192/bjp.120.554.31 5041518

[B11] Bonhoeffer K. (1901). *Die Akuten Geisteskrankheiten der Gewohnheitstrinker*. Jena: Gustav Fischer 9577853

[B12] Bortolotti L. Cox R. Broome M. Mameli M. (2012). “Rationality and self-knowledge in delusion and confabulation: implications for autonomy as self-governance,” in *Autonomy and Mental Disorder* ed. Radoliska L. (New York: Oxford University Press)

[B13] Boyer P. Wertsch J. V. (2009). *Memory in Mind and Culture*. Cambridge: Cambridge University Press

[B14] Brown R. (1957). Sound sleep and sound scepticism. *Australas. J. Philos.* 35 47–53 10.1080/00048405785200041

[B15] Carroll J. S. (1978). The effect of imagining an event on expectations for the event: an interpretation in terms of the availability heuristic. *J. Pers. Soc. Psychol.* 36 1501–1511 10.1037/0022-3514.36.12.1501

[B16] Chapman J. E. (1991). “Use of an Eye-Operated Computer System in Locked-In Syndrome,” in *Proceedings of the Sixth Annual International Conference on Technology and Persons with Disabilities (CSUN’91)* Los Angeles, CA

[B17] Coltheart M. (2006). What has functional neuroimaging told us about the mind (so far)? *Cortex* 42 323–331 10.1016/S0010-9452(08)70358-7 16771037

[B18] Conway M. A. (2005). Memory and the self. *J. Mem. Lang.* 53 594–628 10.1016/j.jml.2005.08.005

[B19] Dement W. Kleitman N. (1957a). The relation of eye movements during sleep to dream activity: an objective method for the study of dreaming. *J. Exp. Psychol.* 53 339–346 10.1037/h0048189 13428941

[B20] Dement W. Kleitman N. (1957b). Cyclic variations in EEG during sleep and their relation to eye movements, body motility and dreaming. *Electroencephalogr. Clin. Neurophysiol.* 9 673–690 10.1016/0013-4694(57)90088-3 13480240

[B21] Dennett D. (1976). Are dreams experiences? *Philos. Rev.* 85 151–171 10.2307/2183728

[B22] Domhoff G. W. (2003). *The Scientific Study of Dreams: Neural Networks, Cognitive Development, and Content Analysis*. Washington: American Psychological Association

[B23] Domhoff G. W. (2007) “Realistic simulation and bizarreness in dream content: past findings and suggestions for future research,” in *The New Science of Dreaming: Content, Recall, and Personality Characteristics* eds Barrett D. McNamara P. (Westport, CT: Praeger Press), Vol.2 1–27

[B24] Doricchi F. Iaria G. Silvetti M. Figliozzi F. Siegler I. (2006). The “Ways” We Look at Dreams: Evidence from Unilateral Spatial Neglect (with an Evolutionary Account of Dream Bizarreness). Experimental Brain Research: Springer-Verlag 10.1007/s00221-006-0750-x 17091297

[B25] Dresler M. Wehrle R. Spoormaker V. Koch S. Holsboer F. Steiger A. (2012). Neural correlates of dream lucidity obtained from contrasting lucid versus non-lucid REM sleep: a combined EEG/fMRI case study. *Sleep* 35 1017–1020 10.5665/sleep.1974 22754049 PMC3369221

[B26] Dunlop C. E. (1977). *Philosophical Essays on Dreaming*. London: Cornell University Press

[B27] Fine C. (2011). Delusions of Gender: How Our Minds, Society, and Neurosexism Create Difference. New York: W. W. Norton and Company Inc

[B28] Flanagan O. (2001). Dreaming Souls: Sleep, Dreams and the Evolution of the Conscious Mind. USA: Oxford University Press

[B29] Foulkes D. (1979). Home and laboratory dreams: empirical studies and a conceptual reevaluation. *Sleep* 2 233–251 232567

[B30] Foulkes D. (1982). A cognitive-psychological model of REM dream production. *Sleep* 5 169–187 6179144 10.1093/sleep/5.2.169

[B31] Foulkes D. (1985). *Dreaming: A Cognitive-Psychological Analysis*. Mahwah: Lawrence Erlbaum

[B32] Foulkes D. (1996). Dream Research 1953–1993. *Sleep* 19 609–624 8958631 10.1093/sleep/19.8.609

[B33] Foulkes D. (1999). *Children’s Dreaming and the Development of Consciousness*. Cambridge: Harvard University Press

[B34] French C. C. Richards A. (1993). Clock This! An everyday example of a schema-driven error in memory. *Br. J. Psychol.* 84 249–253 10.1111/j.2044-8295.1993.tb02477.x

[B35] Garry M. Manning C. G. Loftus E. F. Sherman S. J. (1996). Imagination inflation: imagining a childhood event inflates confidence that it occurred. *Psychon. Bull. Rev*. 3 208–214 10.3758/BF03212420 24213869

[B36] Gregory W. L. Cialdini R. B. Carpenter K. M. (1982). Self-relevant scenarios as mediators of likelihood estimates and compliance: does imagining make it so? *J. Pers. Soc. Psychol*. 43 89–99 10.1037/0022-3514.43.1.89

[B37] Gudjonsson G. H. (1984). A new scale of interrogative suggestibility. *Pers. Individ. Differ.* 5 303–314 10.1016/0191-8869(84)90069-2

[B38] Harley T. A. (2004). Does cognitive neuropsychology have a future? *Cogn. Neuropsychol.* 21 3–16 10.1080/02643290342000131 21038182

[B39] Heaps C. Nash M. (1999). Individual differences in imagination inflation. *Psychon. Bull. Rev.* 6 313–318 10.3758/BF03214120 12199217

[B40] Hobson J. A. (1988). *The Dreaming Brain*. New York: Basic Books

[B41] Hobson J. A. (2002). *Dreaming: An Introduction to the Science of Sleep*. Oxford: Oxford University Press

[B42] Hobson J. A. (2005). In bed with mark solms? What a nightmare! reply to Domhoff. *Dreaming* 15 21–29 10.1037/1053-0797.15.1.21

[B43] Hobson J. A. Pace-Schott E. F. Stickgold R. (2000). Dreaming and the brain: toward a cognitive neuroscience of conscious states. *Behav. Brain Sci.* 23 793–1121 10.1017/S0140525X00003976 11515143

[B44] Horton C. L. (2011). Rehearsal of dreams and waking events similarly improves the quality but not the quantity of autobiographical recall. *Dreaming* 21 181–196 10.1037/a0024860

[B45] Johnson M. K. Kahan T. L. Raye C. L. (1984). Dreams and reality monitoring. *J. Exp. Psychol.* 113 329–344 10.1037/0096-3445.113.3.329 6237167

[B46] Jouvet M. (1999). *The Paradox of Sleep: the Story of Dreaming* ed. Garey L. trans. Cambridge: MIT Press

[B47] Kahan T. L. LaBerge S. (1994). Lucid dreaming as metacognition: implications for cognitive science. *Conscious. Cogn.* 3 246–264 10.1006/ccog.1994.1014

[B48] Kahan T. LaBerge S. (2011). Dreaming and waking: similarities and differences revisited. *Conscious. Cogn.* 20 494–514 10.1016/j.concog.2010.09.002 20933437

[B49] Klein C. (2010a). Images are not the evidence in neuroimaging. *Br. J. Philos. Sci.* 61 265–278 10.1093/bjps/axp035

[B50] Klein C. (2010b). Philosophical issues in neuroimaging. *Philos. Compass* 5 186–198 10.1111/j.1747-9991.2009.00275.x

[B51] LaBerge S. (1981). Directing the action as it happens. *Psychol. Today* 15 48–57

[B52] LaBerge S. (1994). Lucidity research, past and future. *Nightlight* 6 2

[B53] LaBerge S. (2000). Lucid dreaming: evidence and methodology. *Behav. Brain Sci.* 23 962–964 10.1017/S0140525X00574020

[B54] LaBerge S. DeGracia D. J. (2000). “Varieties of lucid dreaming experience,” *Individual Differences in Conscious Experience, * eds Kunzendorf R. G. Wallace B. (Amsterdam: J. Benjamins) 269–307

[B55] LaBerge S. Levitan L. (2004). Lucid Dreaming FAQ, Version 2.3. Available at: [accessed January 16, 2003]

[B56] Levy N. (2006) Consciousness and the Persistent Vegetative State. *Neuroethics and Law Blog*. Available at:

[B57] Mahowald M. W. Schenck C. H. Bornemann M. A. (2005). Sleep related violence. *Curr. Neurol. Neurosci. Rep.* 5 153–158 10.1007/s11910-005-0014-3 15743554

[B58] Malcolm N. (1956). Dreaming and skepticism. *Philos. Rev.* 65 14–37 10.2307/2182186

[B59] Malcolm N. (1957). Dreaming and skepticism: a rejoinder. *Australas. J. Philos.* 35 207–211 10.1080/00048405785200221

[B60] Malcolm N. (1959). *Dreaming* New York: Humanities

[B61] Majaranta P. Raiha K. (2002). “Twenty years of eye typing: systems and design issues,” in *Proceedings of Eye Traction Research and Applications* (New Orleans: ACM press) 15–22

[B62] Merino M. Rivera O. Gómez I. Molina A. Dorronzoro E. (2010). A method of EOG signal processing to detect the direction of eye movements. *Sens. Device Technol. Appl.* 100 18–25 10.1109/SENSORDEVICES.2010.25

[B63] Metcalf K. Langdon R. Coltheart M. (2010). The role of personal biases in the explanation of confabulation *Cogn. Neuropsychiatry* 15 64–94 10.1080/13546800902767703 19736594

[B64] Metzinger T. (2009). *The Ego Tunnel*. New York: Basic Books

[B65] Montangero J. (2012). Dreams are narrative simulations of autobiographical episodes, not stories or scripts. *Rev. Dreaming* 22 157–172 10.1037/a0028978

[B66] Monti M. (2010). Willful modulation of brain activity in disorders of consciousness. *N. Engl. J. Med.* 362 7 10.1056/NEJMoa0905370 20130250

[B67] Ofshe R. J. (1992). Inadvertent hypnosis during interrogation: false confession due to dissociative state; mis-identified multiple personality and the Satanic cult hypothesis. *Inter. J. Clin. Exp. Hypn.* 40 125–156 10.1080/00207149208409653 1399152

[B68] Reinsel R. A. Antrobus J. S. (1992). “Lateralized task performance after awakening from sleep,” in *The Neuropsychology of Sleep and Dreaming* eds Antrobus J. S. Bertini M. (Mahwah: Lawrence Erlbaum)

[B69] Revonsuo A. (1995). Consciousness, dreams, and virtual realities. *Philos. Psychol.* 8 35–58 10.1080/09515089508573144

[B70] Rice H. J. Rubin D. C. (2009). I can see it both ways: first- and third-person visual perspectives at retrieval. *Conscious. Cogn.* 18 877–890 10.1016/j.concog.2009.07.004 19692271 PMC2784183

[B71] Rosen M. Sutton J. (2013). *“Self-Representation and Perspectives in Dreams*,” forthcoming in Philosophy Compass

[B72] Schatzman M. Worsley A. Fenwick P. (1988). “Correspondence during lucid dreams between dreamed and actual events,” in *Conscious* Mind, Sleeping Brain. Perspectives on Lucid Dreaming eds Gackenbach J. LaBerge S. (New York: Plenum Press)

[B73] Schechtman M. (1994). The truth about memory. *Philos. Psychol.* 7 3–18 10.1080/09515089408573107

[B74] Scheid W. (1934). Zur pathopsychologie des korsakow syndroms. *Z. Neurol. Psychiatr.* 151 346–369 10.1007/BF02865465

[B75] Schenck C. H. Bundlie S. L. Patterson A. Mahowald M. (1987). Rapid eye movement sleep behavior disorder: a treatable parasomnia affecting older adults. *JAMA* 257 1786–1789 10.1001/jama.1987.03390130104038 3820495

[B76] Schonbar R. A. (1965). Differential dream recall frequency as a component of “Life-Style”. *J. Consult. Psychol.* 29 465–474 10.1037/h0022464 5318262

[B77] Schredl M. (2002–2003). Factors influencing the gender difference in dream recall frequency. *Imagin. Cogn. Pers.* 22 33–39 10.2190/JR55-WYC2-1GC0-023D

[B78] Schredl M. Neurenberg C. Weiler S. (1996). Dream recall, attitude towards dreams and personality. *Pers. Indiv. Differ.* 20 613–618 10.1016/0191-8869(95)00216-2

[B79] Schwartz S. Maquet P. (2002). Sleep imaging and the neuropsychological assessment of dreams. *Trends Cogn. Sci.* 6 23–30 10.1016/S1364-6613(00)01818-0 11849612

[B80] Shapiro B. Alexander M. Gardner H. Mercer B. (1981). Mechanisms of confabulation. *Neurology* 31 1070–1076 10.1212/WNL.31.9.1070 7196527

[B81] Snyder F. (1970). “The phenomenology of dreaming,” in *The Psychodynamic Implications of the Physiological Studies on Dreams* eds Madow L. Snow L. (Springfield, IL: Thomas) 124–151

[B82] Snyder F. Karacan I. Tharp V. Scott J. (1968). Phenomenology of REM dreaming. *Psychophysiology * 4 375

[B83] Sosa E. (2005). Dreams and philosophy. *Proceedings and Addresses of the American Philosophical Association* 79 7–18

[B84] Spadafora A. Hunt H. (1990). The multiplicity of dreams: cognitive-affective correlated of lucid, archetypal and nightmare dreaming. *Percept. Mot. Skills* 71 627–644 10.2466/pms.1990.71.2.627 2251094

[B85] Spiegel D. (1995). “Hypnosis and Suggestion,” in *Memory Distortion: How Minds, Brains and Societies Reconstruct the Past* ed. Schacter D. E. (Cambridge, MA: Harvard University Press) 129–149

[B86] Sutton J. (1998). *Philosophy and Memory Traces: Descartes to Connectionism*. Cambridge: Cambridge University Press

[B87] Sutton J. (2003). “Memory,” in *Stanford Encyclopedia of Philosophy* ed. Zalta E. N. Summer 2004Edn Available at:

[B88] Sutton J. Harris C. Barnier A. (2010). “Memory and cognition,” in *Memory: Theories, Histories, Debates* eds Radstone S. Schwarz B. (USA: Fordham University Press) 209–226; 488–493

[B89] Tholey P. (1983). Relation between dream content and eye movements tested by Lucid dreams. *Percept. Mot. Skills* 56 875–878 10.2466/pms.1983.56.3.875 6877973

[B90] Whalen J. E. Nash M. R. (1996). “Hypnosis and dissociation: theoretical, empirical, and clinical perspectives,” in *Handbook of Dissociation: Theoretical, Empirical, and Clinical Perspectives* eds Michelson L. Ray W. (New York: Plenum) 191–206 10.1007/978-1-4899-0310-5_9

[B91] Whitty C. Lewin W. (1961). A Korsakoff syndrome in the post-cingulectomy confusional state. *Brain* 83 648–653 10.1093/brain/83.4.648 13784970

[B92] Windt J. M. Metzinger T. (2007). “The philosophy of dreaming and self-consciousness: what happens to the experiential subject during the dream state?,” in *The New Science of Dreaming* eds Barrett D. McNamara P. (Westport, CT: Praeger Imprint/Greenwood Publishers)

[B93] Wittgenstein L. (1958). *Philosophical Investigations* 2nd Edn eds Anscornbe G. Rhees E. M. trans R. Anscornbe G. E. M. (Oxford:Blackwell).

